# Using Functional Magnetic Resonance Imaging to Describe Pain Pathways in the ‘Oldest Old’: A Case Study of a Healthy 97-year-old Female

**DOI:** 10.4172/2167-0846.1000111

**Published:** 2012-08-27

**Authors:** Todd Monroe, Andrew Dornan, Michael A. Carter, Ronald L. Cowan

**Affiliations:** 1 Vanderbilt University School of Nursing, Vanderbilt University Institute of Imaging Science, Nashville, Tennessee, USA; 2 Vanderbilt Psychiatric Neuroimaging Program, Vanderbilt University School of Medicine, Nashville, Tennessee, USA; 3 The University of Tennessee Health Science Center College of Nursing, Memphis Tennessee USA; 4 Vanderbilt Addiction Center, Vanderbilt Psychiatric Neuroimaging Program, Vanderbilt University School of Medicine, Nashville, Tennessee, USA

## Introduction

The prevalence of painful medical conditions increases with age. Pain differences in older adulthood are of special concern because we do not know how brain changes in healthy aging may alter the sensory and affective response to pain. Over the last two decades, neuroimaging studies have described interconnected brain regions that mediate pain processing. In particular, imaging techniques have been used to describe brain activation in networks of structures comprising the lateral and medial pain systems [1–3]. The lateral and medial pain networks are generally associated with the sensory-discriminative and affective-motivational dimensions of pain respectively. Key structures that are associated with the lateral pain network include specific nuclei in the thalamus and primary (S1) and secondary somatosensory (S2) cortex while different but specific nuclei in the thalamus, as well as regions in the insular and cingulate cortices are associated with the medial pain network [4,5].

The functional Magnetic Resonance Imaging Blood Oxygenation Level Dependent (fMRI BOLD) method indirectly measures regional brain physiology [6]. The BOLD signal relies on neural activity-dependent changes in blood deoxyhemoglobin levels [6]. When nerve cells are active they recruit excess oxygenated hemoglobin relative to the amount necessary for the specific metabolic task resulting in lower deoxygenated hemoglobin levels (washout effect), which can be detected by the fMRI sequence [6]. Thus, increased neuronal activity results in increased BOLD signal. fMRI can then be used to assay activity in brain pain pathways during a standardized experimental pain paradigm [7,8]. fMRI has been used to study pain in older adults [7,9], but neither of these studies included people over the age of 85.

## Case Report

The fastest growing age group in the population is between 85 and 96 years old [10]. The current paper demonstrates the feasibility of using psychophysical and neurophysiological methods to study pain in people over the age of 85- often referred to as the ‘oldest old’ [11]. Here we report a case that is part of a larger study examining the psychophysical and neurophysiological response to experimental thermal pain in older women with and without dementia.

### Demographics

The participant was a 97-year old English-speaking female residing in an independent senior living facility in the United States. Her normal activities include going for walks, painting, or shopping with friends or family. She had no history of heart or lung disease, substance abuse/dependence, diabetes, and denied current, chronic, or pain that interferes with normal activities of daily life. She was currently being treated for mild hypertension and serum elevated cholesterol. Medications included atorvastatin, aspirin, alondrate, bisoprolol and Benadryl anti-itch cream. Previous surgical procedures included foot surgery at the age of 71 and a lumpectomy at the age of 56. On physical examination her blood pressure was 126/80 mm/Hg, her respiratory rate was 18 breaths per minute, her pulse was 80 beats per minute, and her body mass index was 25.5.

## Methods

### Pretesting procedure

After securing Institutional Review Board (IRB) approval from the Vanderbilt University IRB, we initially screened the participant and a family member over the phone. After meeting basic inclusion/exclusion criteria (≥ 65 years of age, no unstable medical conditions, not an insulin dependent diabetic, no history of chronic pain, not taking opioids, no movement disorder, and not claustrophobic), a consenting/enrollment visit was scheduled at the participant’s home. The enrollment visit lasted approximately 1 hour and included several questionnaires. The Mini Mental State Exam (MMSE) [12] was given to screen for baseline cognitive ability. The participant was found to be cognitively intact with an MMSE score of 28/30. Because this participant was part of a larger study examining pain in people with dementia, the University of San Diego Brief Assessment of Cognitive Capacity (UBACC) [13] was administered to confirm the subject’s capacity to provide self-consent, which she did.

### Psychophysical and fMRI procedures

Two-weeks following the enrollment visit, the participant arrived at the Vanderbilt University Institute of Imaging Science (VUIIS). The experiment was scheduled over 21/2 hours including time for breaks. First, a series of questionnaires examining depression, anxiety, and pain were collected. Psychophysical testing lasting 45 minutes and was conducted using the Medoc Pathway Pain and Sensory Evaluation System [14]. Next, the participant was asked to rate varying thermal pain stimuli. Starting at a baseline temperature of 30°C [15], incremental thermal stimuli were delivered at a rate of 4°C/sec to the thenar imminence of the right hand. Three trials of each temperature were measured and the average recorded. Temperatures used were ones rated by the participant as “Just Noticeable Warmth” (JNW)”, “Weak Pain (WP)” to a maximum of “Moderately Painful (MP)” [7]. After resting for 10–15 minutes, the thermal stimuli rated by the participant were administered during fMRI procedures lasting approximately 25 minutes. Complete visual and auditory monitoring of the participant was maintained throughout and a family member was present for all in-scanner data collection procedures. Prior to MRI procedures the patient verified that she had not taken any medications for pain in the previous 24 hours.

### fMRI acquisition

Images were acquired with a Philips 3 T Intera Achieva MRI scanner (Philips Medical Systems, Andover, MA) according to routine methods. In each 240 s functional run, 28 field echo EPI (162 dynamics, 4.50 mm slice thickness with 0.45 mm gap, 2s TR, 35 ms TE, 79° (flip angle, FO =240, matrix=128×128) scans were acquired. A standard whole-brain 3-D anatomical T1-weighted/TFE (with SENSE coil) scan was acquired for alignment to enhance accuracy [16].

### fMRI preprocessing

Data were analyzed using SPM8 (Wellcome Department of Cognitive Neuroscience, London, UK) using the General Linear Model (GLM). The functional data were pre-processed, normalizing into MNI space using SPM’s 152 average T1 template (Montreal Neurological Institute). Normalized functional images were smoothed with a full-width half maximum (FWHM) 8 mm Gaussian kernel and motion corrected prior to statistical analysis.

Regions of Interest (ROIs) were determined *a priori* from those comprising the pain matrix--thalamus [thalamic nuclei function in either the lateral (Ventro Posterior Lateral, VPL) or medial (dorsomedial, DM) pathway], primary (S1) and secondary (S2) somatosensory cortex (lateral pathway) and insular and cingulate cortices (medial pathway) [4,5].

### fMRI data analysis

First level T-maps were generated in SPM 8 using standard GLM approaches to contrast brain activation during combined “pain” (JNW+WP+MP) versus baseline. Using a WFU pick atlas, we generated an a priori ROI mask. Voxels were considered significantly activated if they met or exceeded a Family Wise Error corrected threshold of p<0.05, based on a cluster extent threshold of 5. Gray matter volume in selected ROIs was measured using the MarsBaR and easy volume tools within SPM8. Using SPSS 18.0 (IBM Corporation, Somers, NY), a Spearman’s Rho was performed between gray matter volume and percent BOLD signal change.

## Results

### Psychophysical results

The average temperatures at which the participant reported the sensation of pain ranged from 36°C for JNW to 41°C for WP, and 45°C for MP. To capture the affective dimension associated with the pain sensations, the subject provided an affective (unpleasant) pain report using a 0–20 affective pain rating scale [7]. The subject verbally rated the ‘unpleasantness’ associated with JNW, WP, and MP as 0/20, 2/20, and 5/20 respectively.

### fMRI results

**Figure 1:**
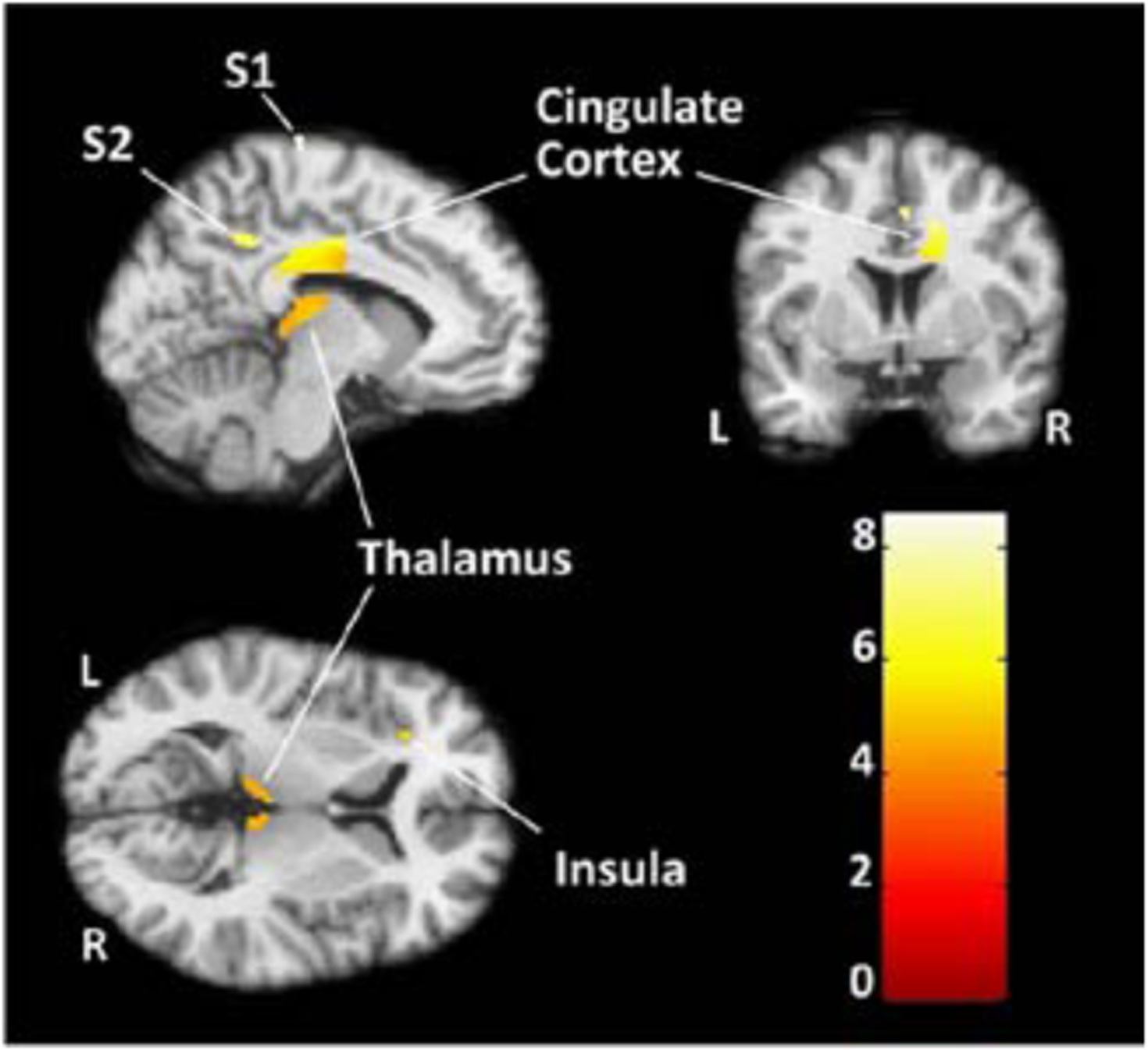
Activation to combined pain conditions (WP+MP) versus baseline in a *priori* brain regions comprising the medial and lateral pain matrix (Family Wise Error [FWE] corrected, P<0.05). Color graph indicates *T*-score intensities. S1=Primary somatosensory cortex, S2=Secondary somatosensory cortex, WP=Week pain; MP=Moderate pain.

fMRI results reveal signifiscant activation in all *a priori* pain related brain regions comprising the pain network systems including the primary and secondary somatosensory, insular, and cingulate cortices (Figure 1). No significant correlation (Spearman’s Rho=−0.300, p=0.624) was found between regional percent BOLD signal change and regional gray matter volume (Table 1).

## Discussion

Including older adults in neurophysiological research is critical in improving our knowledge about brain systems because of the normal changes that occur within the aging human body. Here, we demonstrate that fMRI can be used successfully in the oldest old. Furthermore, the psychophysical and fMRI data presented demonstrate that the sensory/discriminative and affective/motivational dimensions of pain are maintained while significant brain activation patterns associated with acute experimental pain remain robust in the oldest old. This would suggest that the pain experience does not diminish with aging in the oldest of old. As the population continues to age, psychophysical and neurophysiological studies examining both the neurobiology of pain and the response to interventions to pain in older adults will be critical to developing future pain treatments.

## Figures and Tables

**Table 1:** Percent BOLD signal change and gray matter volume in selected ROIs.

Region of Interest	Percent BOLD Signal Change	Gray Matter Volume (ml)
Cingulate Cortex	2.00	16.4
Insula	1.96	11.95
S2	2.25	1.63
S1	2.20	4.16
Thalamus	1.08	3.64
